# MiR-486-5p Serves as a Good Biomarker in Nonsmall Cell Lung Cancer and Suppresses Cell Growth With the Involvement of a Target PIK3R1

**DOI:** 10.3389/fgene.2019.00688

**Published:** 2019-07-26

**Authors:** Fei Tian, Jun Wang, Tinglan Ouyang, Na Lu, Jiafeng Lu, Yanting Shen, Yunfei Bai, Xueying Xie, Qinyu Ge

**Affiliations:** ^1^State Key Lab of Bioelectronics, School of Biological Science and Medical Engineering, Southeast University, Nanjing, China; ^2^Department of Thoracic Surgery, Jiangsu Province People’s Hospital and the First Affiliated Hospital of Nanjing Medical University, Nanjing, China; ^3^Center of Reproduction and Genetics, Affiliated Suzhou Hospital of Nanjing Medical University, Suzhou Municipal Hospital, Suzhou, China

**Keywords:** nonsmall cell lung cancer, miR-486-5p, PIK3R1, biomarker, proliferation

## Abstract

MicroRNAs are a class of noncoding RNAs that can be involved in the regulation of gene expression in cancers, including lung cancer. Our previous research has shown that miR-486-5p is one of the most downregulated microRNAs in tissue and serum samples of lung cancer as a good diagnostic biomarker. The objective of this study is to investigate the roles of miR-486-5p in the progression of lung cancer. In this study, miR-486-5p was further validated to be significantly downregulated in additional nonsmall cell lung cancer (NSCLC) tissue, serum, and cell samples by quantitative reverse transcription-polymerase chain reaction (RT-PCR), and the expression level of miR-486-5p was significantly associated with clinical phenotype of NSCLC. The PIK3R1 gene was confirmed to be a direct target of miR-486-5p by dual-luciferase reporter assay, and the expression level of miR-486-5p was inversely correlated with that of PIK3R1 in tumor tissues (*r* = −0.774, *p* < 0.01). Overexpressed miR-486-5p effectively inhibited cell proliferation and invasion and successfully induced apoptosis *in vitro*. PIK3R1 was involved in the suppression of miR-486-5p on cell growth. It can be concluded that miR-486-5p may act as a tumor suppressor contributing to the progression of NSCLC, and miR-486-5p would be a diagnostic and prognostic biomarker and a potential therapeutic target for lung cancer.

## Introduction

Lung cancer is the most common incident cancer and the leading cause of cancer-related death, with 733,300 new cases and 610,200 deaths in China in 2015 (Chen et al., 2016). Lung cancer patients were frequently diagnosed at advanced stages, resulting in a high mortality. Nonsmall cell lung cancer (NSCLC) is the most common subtype of lung cancer, including two main histologic subtypes: lung squamous cell carcinoma (SCC) and lung adenocarcinoma (AC) (Travis et al., 2015). In recent years, the early diagnosis and targeted therapy of lung cancer attracted more attention, and studies shown that molecular biomarkers have been discovered in different tumors and could be well applied in the early diagnosis, prognosis, and therapy of lung cancer (Zhang et al., 2016a; Best et al., 2017).

MicroRNAs (miRNAs) are endogenous noncoding small RNAs (∼22 nt) that modulate the activity of mRNA by hybridizing to complementary sequences in the 3’-untranslated region (UTR) of specific targets (Bartel, 2004; Kloosterman and Plasterk, 2006). A lot of studies have demonstrated that miRNAs could participate in various cell biological processes, including cell growth, differentiation, and cell apoptosis (Ambros, 2003; Kloosterman and Plasterk, 2006; Stefani and Slack, 2008). Moreover, studies have shown that miRNAs are frequently dysregulated in cancers, including NSCLC (Calin and Croce, 2006; Esquela-Kerscher and Slack, 2006; Kumar et al., 2008). Recently, miR-486-5p has been found to function as a potential suppressive gene in different cancers, including lung cancer (Peng et al., 2013; Wang et al., 2014; Liu et al., 2016; Zhang et al., 2016b). Coincidentally, our previous study found aberrant miR-486-5p expression in both tissue and serum samples of NSCLC by Hiseq2500 sequencing; miR-486-5p might be an early diagnostic biomarker for NSCLC (Tian et al., 2016). However, the opposite expression levels and roles of miR-486-5p in lung cancer were identified in different reports (Yu et al., 2010; Tan et al., 2011; Jin et al., 2017; Sromek et al., 2017). Therefore, we need to further evaluate and validate the biological function of miR-486-5p in the progression of NSCLC in this study and whether miR-486-5p have an important role in NSCLC by targeting other functional genes?

In the current study, the expression level of miR-486-5p was further confirmed to be significantly lower in NSCLC tissue, serum, and cell lines than in the corresponding controls, and low expression of miR-486-5p was significantly inversely correlated with advanced tumor tumor-mode-metastasis(TNM) stage and larger tumor size in NSCLC tissue. Overexpressed miR-486-5p significantly restrained proliferation and invasion of A549 and H1299 cells and induced cell apoptosis. PIK3R1 was involved in the suppression of miR-486-5p on cell growth. Therefore, miR-486-5p may act as a potent tumor suppressor of cellular growth and migratory capacity in NSCLC.

## Materials and Methods

### Clinical Tissue and Serum Specimens

A total of 36 paired surgical cancerous, normal tissue and serum samples were obtained from NSCLC patients, and the 36 normal serum samples were obtained from healthy volunteers. Another 39 tumor tissue samples were also collected for clinical pathology analysis by qRT-PCR (**Supplementary Table 1**). All samples mentioned were obtained from Jiangsu Province People’s Hospital with informed consent. This study was approved by the Ethics Committee of Jiangsu Province People’s Hospital. All tissue and serum specimens were stored at −80°C for subsequent RNA extraction.

### Cell Culture

The human NSCLC cell line A549, H1299, and normal pulmonary epithelial cell line BEAS-2B and human renal epithelial cell line 293T were obtained from the Cell Bank, China Academy of Sciences (Shanghai, China). Cells were cultured in DMEM medium (Corning, USA) supplemented with 10% (v/v) fetal bovine serum (Biological Industries, Israel) at 37°C in 5% CO_2_ atmosphere.

### RNA Extraction and Quantitative RT-PCR

Tissue and cell RNAs were isolated using mirVana^™^ miRNA isolation Kit (Ambion, USA); serum RNA was isolated according to the protocol of miRNeasy Serum/Plasma Kit (Qiagen, Germany). MiRNA and mRNA analysis were performed by qRT-PCR using PrimeScript^™^ II reverse transcriptase (Takara, Japan) and SYBR Premix Ex Taq^™^ (Takara, Japan) according to the manufacturer’s protocols. Relative quantification of miR-486-5p and PIK3R1 were obtained by normalization to U6 snRNA and β-Actin, respectively. The qRT-PCR experiments were repeated at least three times. The expression levels of miR-486-5p and PIK3R1 were calculated by ΔΔCt, normalized with ΔCt = AvgCt*_miR-486-5p_* − AvgCt*_U6_* or AvgCt*_PIK3R1_* − AvgCt_β_ *_-Actin_*. The primers used in this study are listed in **Supplementary Table 2**.

### Bioinformatics

The target genes of miR-486-5p were predicted by the intersection of five prediction software packages, microT, miRmap, TargetScan, PicTar, and miRanda, based on starBase v3.0 (Yang et al., 2011; Li et al., 2014). The selected PIK3R1 contained regions in the 3’-UTRs that perfectly matched the seed sequence of miR-486-5p. The Cancer Genome Atlas (TCGA) data were employed to analyze the expression level of miR-486-5p in lung SCC and AC. The *PI3K-Akt signaling pathway* and *nonsmall cell lung cancer pathway* were analyzed in the KEGG database (https://www.kegg.jp/). The Kaplan–Meier plotter was adopted for the prognosis analysis of miR-486-5p in NSCLC (Lanczky et al., 2016; Nagy et al., 2018).

### PIK3R1 3’-UTR Luciferase Reporter Assay

The human PIK3R1 3’-UTR and luciferase reporter construct were synthesized by cloning the human PIK3R1 3’-UTR sequence into a GP-miRGLO report vector (Sangon Biotech, China). The PIK3R1 3’-UTR wild type, mutant type, and a positive control (miR-486-5p inhibitor sponge) on binding sites were directly chemo-synthesized (**Supplementary Table 3**). The synthesized fragment was cloned into GP-miRGLO luciferase report vector at SacI and XhoI sites. The sequence of plasmid was confirmed by DNA sequencing. The 293T cells (5 × 10^5^ per well) were seeded in a 12-well plate the day before transfection, and then cotransfected with firefly luciferase 3’-UTR (GP-miRGLO-PIK3R1), pRL-TK-Renilla-luciferase plasmid (Promega, USA), and miR-486-5p mimics or negative control (NC). After 24 and 48 h, luciferase activity was measured with the Dual-Luciferase Reporter Assay System (Promega, USA), respectively. Firefly luciferase activity was normalized to *Renilla* luciferase activity for transfection efficiency. Experiments were repeated at least three times.

### Lentivector Infection, Transfection, and siRNA Silencing

To force the expression of miR-486-5p in NSCLC cells, the pre-miR-486-5p construct (sequence shown in **Supplementary Table 3**) was packaged with a pGLV2-U6 lentivector packaging plasmid mix (Sangon Biotech, China) in a 293T packaged cell line. The Transdux reagent (System Bioscience, USA) was used for virus transduction, and infected cells were selected by ampicillin (Sigma, USA). Transfection of pre-miR-486-5p expression vector was carried out with Lipofectamine 2000 according to the manufacturer’s instruction (Invitrogen, USA), and transfected A549 and H1299 cells were selected by penicillin and streptomycin (Gibco, USA). Similarly, the lentivector packaging plasmid was inserted PIK3R1 sequence to overexpress PIK3R1.

Two small interfering RNAs (siRNAs) specifically against PIK3R1 (si-739 and si-1178) were designed, and the sequences are shown in **Supplementary Table 3**. Transfections were performed using Lipofectamine 2000 reagent (Invitrogen, USA) following the manufacturer’s protocol with si-PIK3R1 or scrambled sequences. At least three independent experiments were carried out.

### Western Blot

Total proteins (50–100 μg) extracted from cell lines were analyzed by SDS–polyacrylamide gel electrophoresis and were transferred electrophoretically to a PVDF membrane (Millipore, USA). To evaluate the expression of PIK3R1 (p85α), blots were blocked with 5% nonfat milk in Tris-buffered saline and Tween 20 and incubated with a primary rabbit anti-PIK3R1 polyclonal antibody, PAB19524 (Abnova). The polyclonal PAB19524 antibody was produced by immunizing animals with a synthetic peptide corresponding to amino acids at N-terminus of human PIK3R1. Antibody for GAPDH, G8795 (Sigma, USA), was used as a control. The blots were then reprobed with secondary antibody and detected with SuperSignal West Pico Chemiluminescent Substrates System (Pierce, USA).

### Cell Proliferation, Transwell, and Annexin V Apoptosis Assay

Transfected cells were measured for cell proliferation with Cell Counting Kit-8 (CCK-8, Dojindo, Japan) after incubating for 24, 48, and 72 h in a 96-well microplate (Corning, USA). Each well was added with 5 μl CCK-8 and 100 μl fresh medium, then incubated for 2 h. The absorbance was measured at 450 nm by Multiskan FC (Thermo Fisher, USA). The A549 and H1299 cells without any transfection were analyzed as the blank controls, and the cells transfected with empty vector were the NC.

To determine cell invasion and migration, cells were plated in medium with 1% serum in the top chamber of a Transwell (Corning, USA) after transfection. The bottom chamber contained standard medium with 20% fetal bovine serum. After 72-h incubation, the cells that had migrated to the lower surface of the membrane were fixed with cold ethyl alcohol, stained with 0.1% crystal violet, and photographed under a microscope. Cell numbers were counted under a light microscope at ×400 magnification. Experiments were carried out at least three times.

Cells were stained with annexin V fluorescein isothiocyanate (V-FITC) and propidium iodide (PI) using the Annexin V-FITC Kit (Beckman Coulter, USA) for flow cytometric analysis (FACSCalibur, BD Bioscience, USA). The apoptotic index was calculated as the percentage of annexin V+/PI cells.

### Statistical Analysis

The differences of two groups were analyzed using paired *t*-test with one-tailed *p* value. One-way ANOVA was used to determine the difference in miR-486-5p expression in three groups of different clinicopathological factors. Pearson correlation analysis was used to determine the correlation between miR-486-5p and PIK3R1 expression with two-tailed *p* value. In all cases, *p* < 0.05 was considered statistically significant difference.

## Results

### MiR-486-5p Was Significantly Downregulated and Associated With Clinical Phenotype and Survival Data in NSCLC

**Figure 1:**
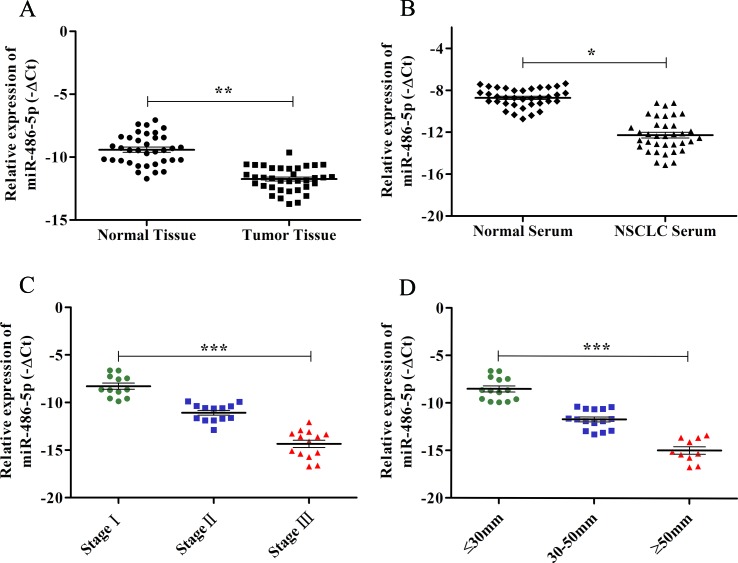
The expression levels of miR-486-5p and correlations with clinical data in NSCLC samples by qRT-PCR. **(A)** The relative expression levels of miR-486-5p were calculated by −ΔCt in corresponding tumor tissue and normal tissue. **(B)** The relative expression levels of miR-486-5p in NSCLC patients’ serum and normal serum by −ΔCt method. **(C)** The relative expression levels of miR-486-5p were significantly lower in stages II and III compared to stage I (*p* < 0.001). **(D)** The expression levels of miR-486-5p were significantly lower in the larger tumor (*p* < 0.001). *p < 0.05, **p < 0.01, ***p < 0.001.

A previous study has reported that miR-486-5p was significantly downregulated in NSCLC tissue and serum compared to the normal tissue and serum, and the Hiseq 2500 sequencing and qRT-PCR results showed good consistency (Tian et al., 2016). In this study, we further validated the low expression of miR-486-5p in additional 36 paired tissue (*p* < 0.01, **Figure 1A**) and serum samples (*p* < 0.05, **Figure 1B**) of NSCLC compared with corresponding controls by qRT-PCR. The TCGA data showed miR-486-5p was significantly lower expressed in lung SCC (322 samples, *p* < 0.01) and AC tissue (430 samples, *p* < 0.01) compared to the controls (**Supplementary Figure 1**). These results illustrated that the downregulated miR-486-5p might be associated with NSCLC carcinogenesis as a good diagnostic biomarker.

**Figure 2:**
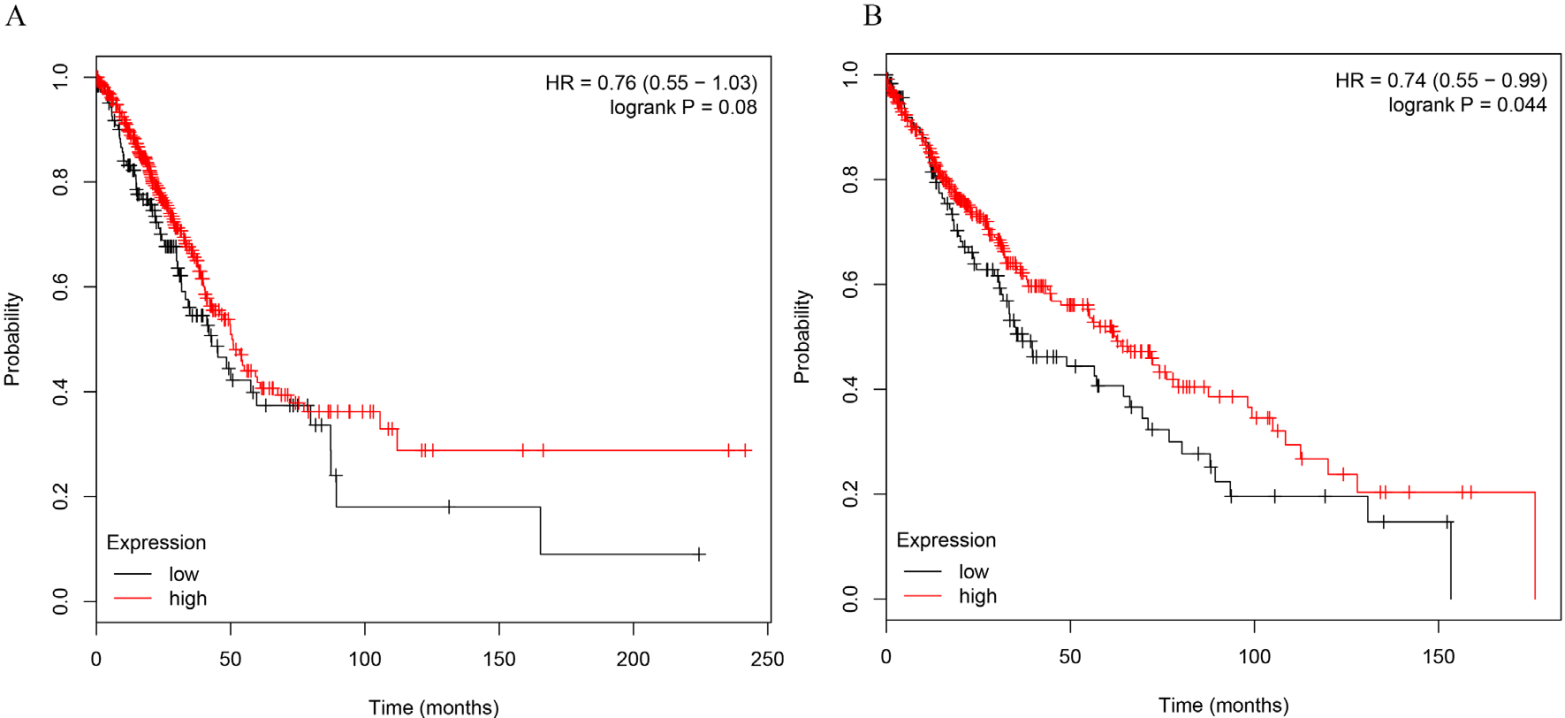
The correlations of miR-486-5p levels and survival months in NSCLC patients. **(A)** No significant correlation between miR-486-5p expression and prognosis data in AC patients (504 samples, *p* = 0.08). **(B)** SCC patients with low expression of miR-486-5p had worse prognosis (472 samples, *p* = 0.044).

To further determine the relations of clinicopathological factors (**Supplementary Table 1**) with miR-486-5p expression, we evaluated the levels of miR-486-5p in another 39 tumor tissue samples by qRT-PCR. The results revealed that the expression level of miR-486-5p was statistically decreased in stages III and II compared to stage I (*p* < 0.001, **Figure 1C**). Moreover, the relative expression level of miR-486-5p was significantly lower in tumor with size ≥5 or 30–50 mm than in size ≤30 mm (*p* < 0.001, **Figure 1D**). Low expression of miR-486-5p was inversely correlated with advanced tumor TNM stage and larger tumor size in NSCLC tumor tissues. However, there were no significant correlations in miR-486-5p expression levels and patients’ age, gender, smoking status, or histological type (**Supplementary Table 1**). From the Kaplan–Meier plotter, low expression of miR-486-5p had significantly worse prognosis in lung SCC patients (472 samples, *p* = 0.044). However, there was no significance between its expression and prognosis data in lung AC patients (504 samples, *p* = 0.08, **Figure 2**). It could be inferred that the low miR-486-5p expression was closely correlated to the progression and prognosis of NSCLC, and miR-486-5p might be a tumor suppressor in NSCLC.

### PIK3R1 Was a Direct Target of miR-486-5p

**Figure 3:**
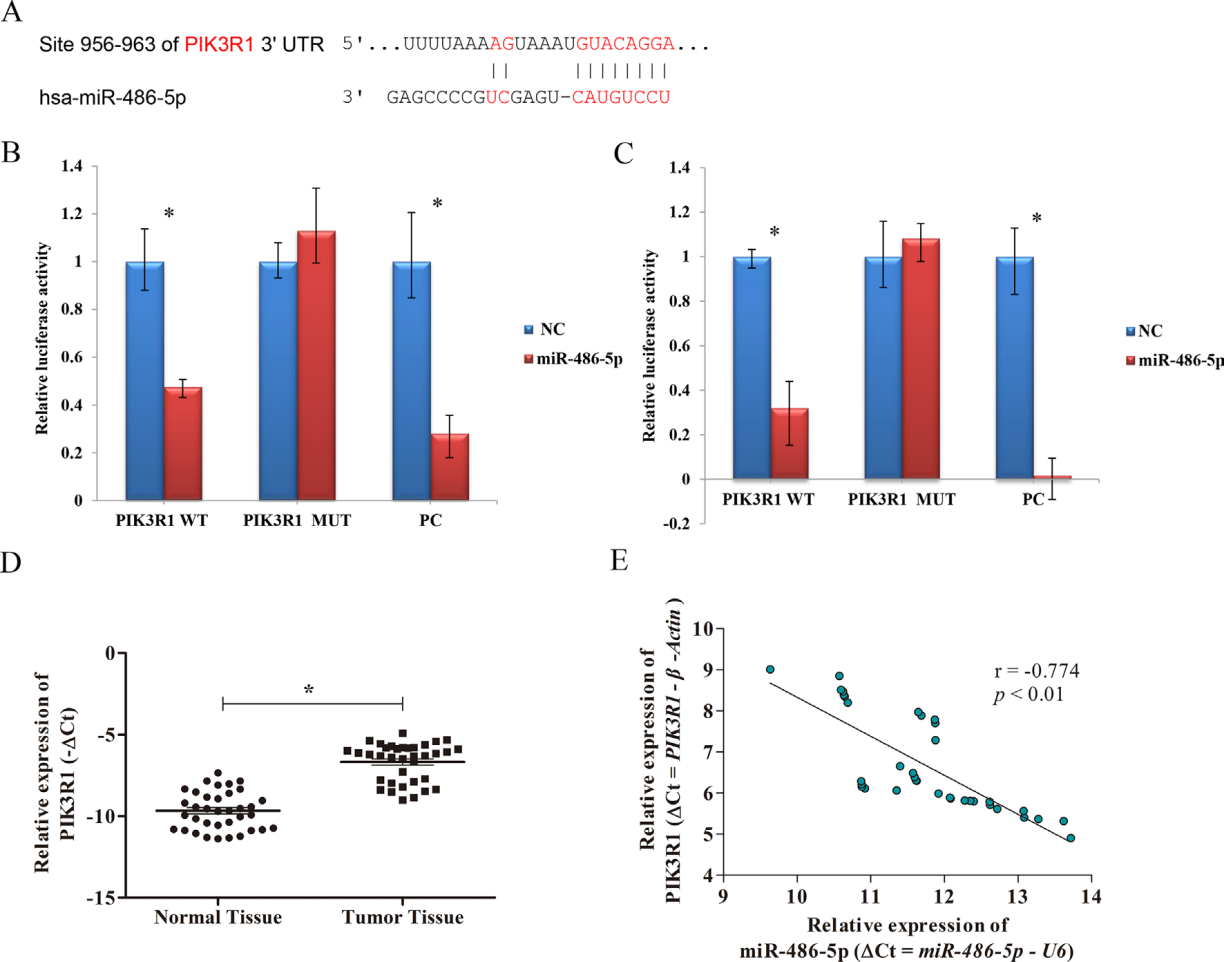
The correlation of PIK3R1 and miR-486-5p expression levels. **(A)** PIK3R1 3’-UTR contains miR-486-5p binding sites. The alignment of the seed region of miR-486-5p and the site of target were indicated in red. **(B**, **C)** The luciferase reporter assay showed that the luciferase activity of PIK3R1-3’-UTR wild type (WT) was significantly reduced in cells expressing miR-486-5p compared with those expressing negative control (NC) (*p* < 0.01), but the luciferase activity of PIK3R1-3’-UTR mutation type (MUT) did not reveal significant changes after 24 h **(B)** and 48 h **(C)**. The positive control (PC) also exhibited reduction of luciferase activity after 24 and 48 h with transfection (*p* < 0.01). **(D)** The expression level of PIK3R1 was upregulated in NSCLC tumor tissue compared to the normal tissue (*p* < 0.05, *n* = 36). **(E)** Upregulation of PIK3R1 was negatively correlated with downregulation of miR-486-5p in NSCLC tissue by ΔCt (*r* = −0.774, *p* < 0.01, *n* = 36). *p < 0.05.

To elucidate the biological mechanism responsible for miR-486-5p as a tumor suppressor, bioinformatics analysis was adopted to search its target genes. Several potential targets of miR-486-5p were identified using microT, miRmap, TargetScan, PicTar, and miRanda programs based on the starBase v3.0. Specifically, the CLIP data were adopted with strict stringency ≥5; the pan-cancer data with six cancer types and above five programs were employed to predict. Totally, three mRNAs were identified, including ST5, PIK3R1, and SRSF3. However, no related papers have reported the regulation of miR-486-5p to the gene ST5 or SRSF3. On the contrary, some studies found the relations of miR-486-5p and PIK3R1 in other cancers: colorectal cancer and hepatocellular carcinoma (Huang et al., 2015; Zhang et al., 2018). Interestingly, miR-486-5p was reported to have different effects on the target PIK3R1 in A549 cell lines (Peng et al., 2013; Gao et al., 2018). Therefore, we preferentially selected PIK3R1 as one potential target of miR-486-5p to validate their relations in lung cancer. In particular, the 3’-UTR of PIK3R1 mRNA perfectly harbored complementary sequences to the miR-486-5p seed sequence (**Figure 3A**).

To further determine whether PIK3R1 could be directly regulated by miR-486-5p, we performed dual luciferase reporter assay. After 24 and 48 h with transfection, the luciferase activity of PIK3R1-3’-UTR wild type was reduced by ∼ 52% and ∼68% in cells expressing miR-486-5p compared to those expressing mimic NC, respectively (*p* < 0.01). However, the luciferase activity did not reveal significant reduction in PIK3R1-3’-UTR mutant type by miR-486-5p. A positive control with binding sites of miR-486-5p showed good consistent result with the wild-type case (**Figures 3B, C**). Therefore, PIK3R1 was validated to be a direct target of miR-486-5p, and miR-486-5p could inhibit PIK3R1 by binding to its 3’-UTR sequence.

To further elucidate the relationship of miR-486-5p and PIK3R1 in clinical NSCLC specimens, qRT-PCR assay found that PIK3R1 was significantly upregulated in NSCLC tissue compared with normal tissue (**Figure 3D**). Moreover, there was a significantly inverse correlation between miR-486-5p and PIK3R1 expression levels in tumor tissue (*r* = −0.774, *p* < 0.01, **Figure 3E**), it revealed that the expression of PIK3R1 might be affected by miR-486-5p in NSCLC.

### Overexpressed miR-486-5p Inhibited PIK3R1 in NSCLC Cell Lines

**Figure 4:**
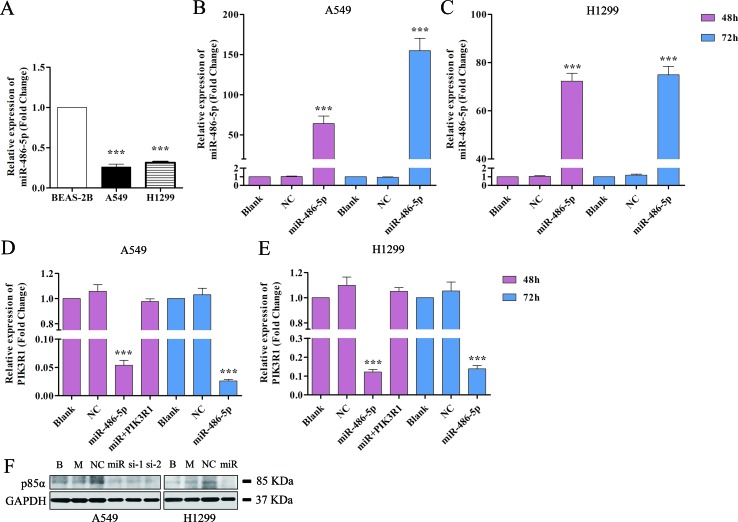
Overexpressed miR-486-5p significantly inhibited the mRNA and protein levels of PIK3R1. **(A)** The fold change of miR-486-5p in A549 and H1299 cells compared to human normal pulmonary epithelial cell lines, BEAS-2B. **(B**, **C)** The relative expressions of miR-486-5p in transfected A549 **(B)** and H1299 **(C)** cell lines were significantly higher compared to the blank (with no transfection) and NC (negative control, transfected with empty vector) after 48 or 72 h with transfection. **(D**, **E)** Overexpression of miR-486-5p led to the reduction of PIK3R1 gene in A549 **(D)** and H1299 **(E)** cell lines after 48 or 72 h transfection. **(F)** The miR-486-5p mimic or two siRNAs dramatically reduced the protein expression of PIK3R1 compared with the controls in A549 or H1299 cells after 48 h transfection. ***p < 0.001, B: blank, M: mock, miR: miR-486-5p, si-1: si-739, si-2: si-1178.

We also found that miR-486-5p was downregulated in NSCLC A549 and H1299 cell lines compared to normal pulmonary epithelial cell lines, BEAS-2B (**Figure 4A**). Therefore, we hypothesized that miR-486-5p might function as a tumor suppressor. To validate that overexpressed miR-486-5p could lead to a reduction of PIK3R1 mRNA, pre-miR-486-5p lentivector was constructed and transfected into NSCLC A549 and H1299 cell lines. The results showed that miR-486-5p had observably higher expression in transfected A549 and H1299 cells than in the corresponding controls (*p* < 0.01) after 48 h, and the expression level of miR-486-5p was higher after 72 h incubation than 48 h in A549 cell line (**Figure 4B**) but not in H1299 (**Figure 4C**). Interestingly, the target PIK3R1 showed lower expression level in the transfected cells compared to the controls, especially after 72 h in A549 cells (**Figure 4D, E**). These results suggested that transfected cells could effectively upregulate miR-486-5p and inhibit PIK3R1 level.

To further determine whether the PIK3R1 protein was dysregulated by miR-486-5p, we transfected A549 cells with miR-486-5p (mimic) and two small interfering RNAs (siRNAs) specifically against PIK3R1 (si-739, si-1178) to inhibit PIK3R1 gene. As shown in **Figure 4F** by Western blot, miR-486-5p (∼54% reduction), si-739 (∼47%), and si-1178 (∼61%) dramatically reduced the expression of PIK3R1 (p85α) after 48 h with transfection. Consistently, miR-486-5p could also effectively reduce the expression of PIK3R1 in H1299 cells (∼57%). Therefore, the results revalidated that miR-486-5p effectively inhibited the translation of PIK3R1.

### PIK3R1 Was Involved in miR-486-5p-Induced Suppression of NSCLC Cell Growth

**Figure 5:**
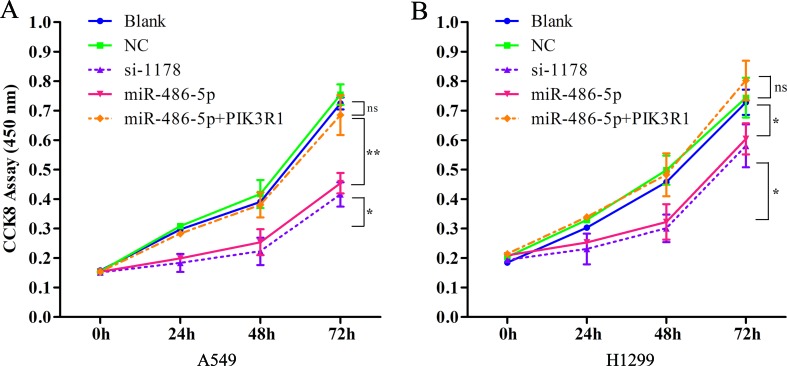
PIK3R1 was involved in miR-486-5p-induced suppression of NSCLC cell proliferation. **(A)** The cell proliferation of A549 cells was evaluated with CCK8 assay with transfecting pre-miR-486-5p, si-1178, or pre-miR-486-5p plus PIK3R1 in 0, 24, 48, and 72 h. The absorbance was measured at 450 nm. The cell proliferation level of miR-486-5p or si-1178 group was significantly lower than the blank or NC after 48 h (*p* < 0.05) and extremely significantly lower after 72 h (*p* < 0.01). However, the pre-miR-486-5p plus PIK3R1 group diminished the effect (ns). **(B)** Similar situation of the cell proliferation in H1299 cell lines with overexpression miR-486-5p and PIK3R1. *p < 0.05, **p < 0.01, ns, not significant.

As the overexpressed miR-486-5p inhibited PIK3R1, the phosphoinositide-3-kinase regulatory subunit 1, which could take part in the PI3K pathway, we sought to determine whether ectopic expression of miR-486-5p had effects on cell metabolism in NSCLC. Interestingly, CCK-8 proliferation assay revealed that forced expression of miR-486-5p significantly reduced cell growth compared to the blank control (with no transfection) and the NC (transfected with empty vector) in A549 (**Figure 5A**) and H1299 cell lines (**Figure 5B**) after 48 h (*p* < 0.05), especially after 72 h in A549 cells (*p* < 0.01). Moreover, si-PIK3R1 (si-1178) mimicked the effect of miR-486-5p overexpression. The blank and NCs had no significant difference; this suggested that cell proliferation was affected by miR-486-5p itself, not by the vector. To evaluate whether dysregulation of PIK3R1 was involved in cell proliferation as a target of miR-486-5p, A549 and H1299 cells were enforced to overexpress miR-486-5p and PIK3R1 at the same time. Compared to the cells with only transfection of miR-486-5p or blank, the cotransfected cells had significantly increased cell growth in both A549 (**Figure 5A**) and H1299 cell lines (**Figure 5B**). These observations suggested that the effects of miR-486-5p on the inhibition of cancer cell proliferation could be diminished by overexpressed PIK3R1. Consistently, the cotransfected cells increased the mRNA levels of PIK3R1 compared to the only overexpressed miR-486-5p cells in both A549 (**Figure 4D**) and H1299 cell lines (**Figure 4E**) after 48 h. In conclusion, miR-486-5p effectively inhibited cell proliferation by targeting PIK3R1.

**Figure 6:**
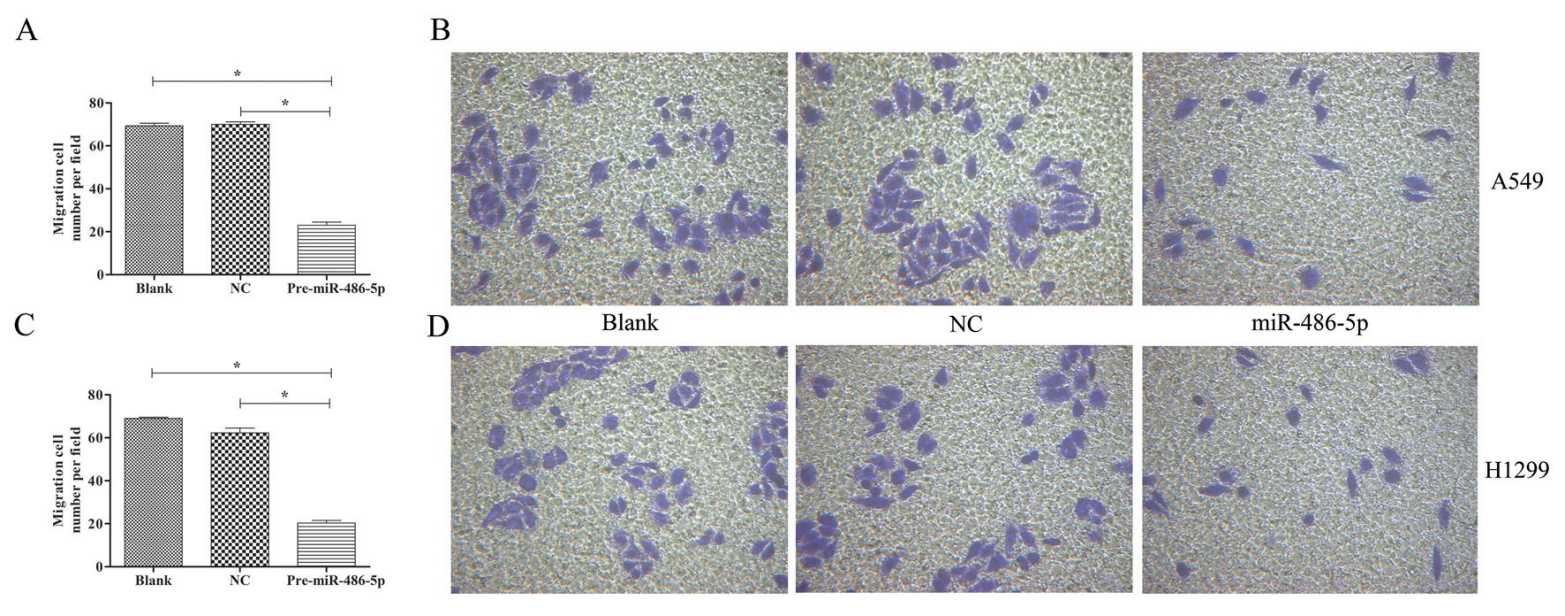
Overexpressed miR-486-5p significantly inhibited A549 and H1299 cell invasion. **(A)** The migrated cell numbers of A549 cells were significantly reduced after 72 h incubation by Transwell assay. The number of cells was calculated with crystal violet staining. **(B)** Representative pictures of Transwell assay in A549 cell lines. Similar results were obtained in three independent experiments. **(C)** The migrated cell numbers of H1299 cells after 48 h incubation. **(D)** Representative pictures of Transwell assay in H1299 cell lines. *p < 0.05.

**Figure 7:**
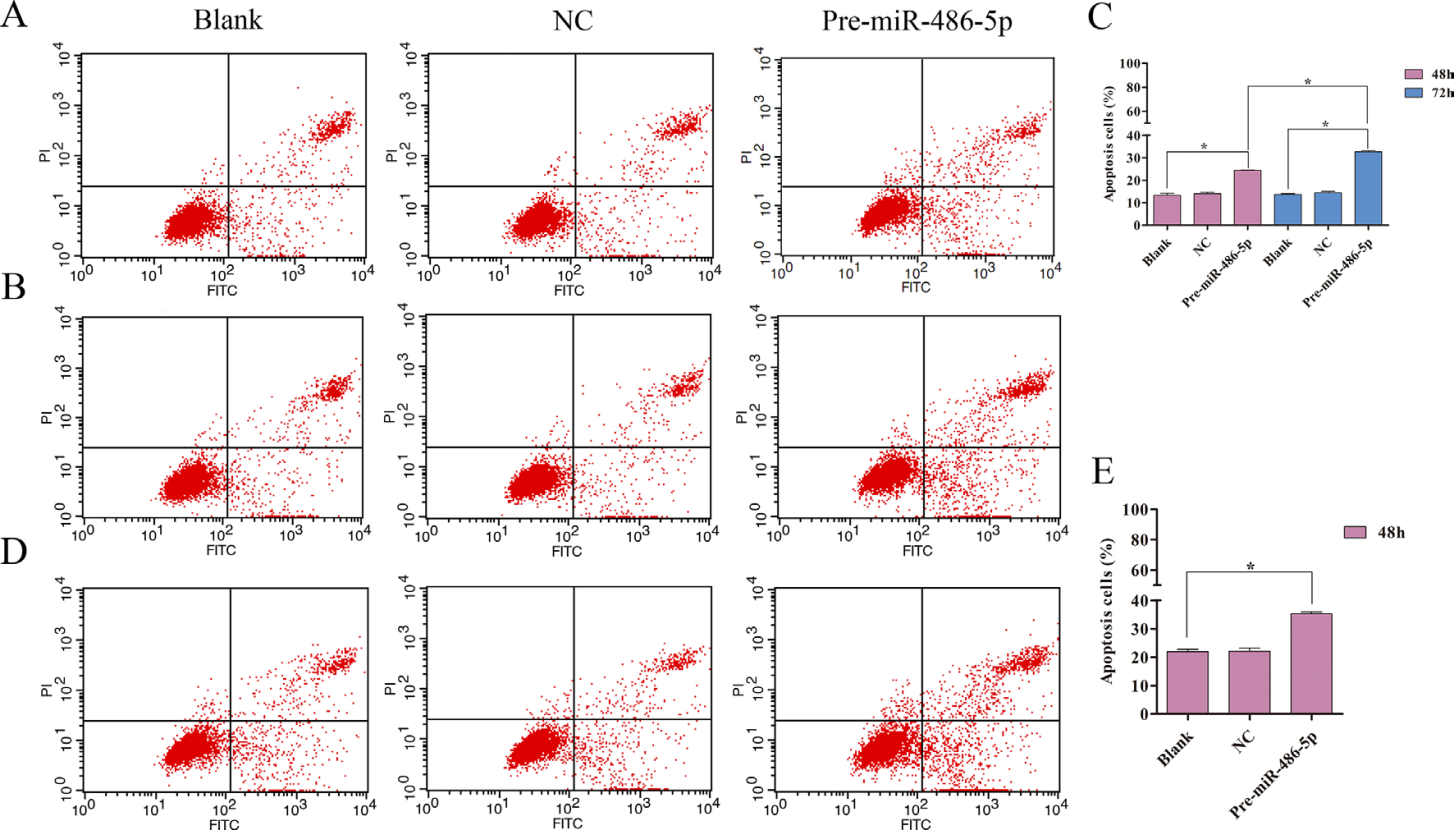
Overexpressed miR-486-5p significantly induced A549 and H1299 cell apoptosis. **(A**, **B)** A549 cells were stained with annexin V fluorescein isothiocyanate (V-FITC) and propidium iodide (PI) after 48 h **(A)** and 72 h **(B)** incubation. One representative experiment was shown, and similar results were obtained in three independent experiments. **(C)** The percentage of A549 apoptosis cells were shown after 48 and 72 h incubation, respectively. **(D)** One representative experiment was shown in H1299 cell apoptosis after 48 h with transfection. **(E)** The percentage of H1299 apoptosis cells were shown after 48 h incubation. *p < 0.05.

To further explore cell invasion abilities with miR-486-5p overexpression, Transwell assay was performed and found that increased miR-486-5p could effectively suppress the migration and invasion of A549 (**Figures 6A, B**) and H1299 cell lines (**Figures 6C, D**) after 48 h. Furthermore, annexin V apoptosis assay showed that the pre-miR-486-5p case had a higher apoptosis rate compared with the controls in A549 (**Figures 7A–C**) and H1299 cell lines (**Figures 7D–E**), especially after 72 h in A549 cells (**Figure 7B**). The empty vector control (NC) did not reveal induced apoptosis on cells, which also validated that the effects were from the overexpressed miR-486-5p, not by the vector.

## Discussion

Our previous study demonstrated that miR-486-5p was significantly downregulated both in tissue and serum samples of NSCLC; it revealed that miR-486-5p might function as a tumor suppressor (Tian et al., 2016). We further validated that miR-486-5p was downregulated in additional 36 paired tissue and serum samples. Moreover, a lower level of miR-486-5p was found in NSCLC A549 and H1299 cell lines compared to normal pulmonary epithelial cell lines. MiR-486-5p is located on chromosome 8p11.21, which is one of the most frequent genomic deletion regions containing potential tumor-suppressive genes in various tumors (Oh et al., 2011). Allelic loss of the genomic region may be responsible for the downregulation of miR-486-5p. Furthermore, miR-486-5p is located in a CpG island on chromosome 2q35; epigenetic silencing by DNA methylation modification may also lead to miR-486-5p downregulation (Wang et al., 2014). Interestingly, some papers had reported the role of miR-486-5p on diagnosis (Yu et al., 2010; Tan et al., 2011; Jin et al., 2017; Sromek et al., 2017) and prognosis (Hu et al., 2010) in lung cancer. Nevertheless, the opposite expression levels and roles of miR-486-5p in lung cancer were found in these related reports. Therefore, we intended to further validate the effects of aberrant miR-486-5p on cell growth and the regulatory pathway in NSCLC. Moreover, several studies have shown that miR-486-5p had important functions by targeting different genes in NSCLC. Pang et al. (2014) validated that downregulated miR-486-5p and upregulated eIF4E led to the overexpression of Pim-1 kinase, a critical survival signaling factor in NSCLC. Peng et al. (2013) recently reported that miR-486 directly targeted insulin growth factor signaling and functioned as a potent tumor suppressor of lung cancer both *in vitro* and *in vivo*. In this study, PIK3R1 was identified as a target of miR-486-5p, and we intended to further evaluate whether PIK3R1 was involved in the suppression of miR-486-5p on NSCLC.

The PIK3R1 gene is localized on 5q13.1. PIK3R1 gene (phosphoinositide-3-kinase regulatory subunit 1) encodes the 85-kD regulatory subunit p85α, a well-accepted subunit of class IA PI3K (Zhao and Vogt, 2008; Mellor et al., 2012). PIK3R1 is the 11th most commonly mutated gene across cancer lineages in TCGA database (Cerami et al., 2012). Although several studies have shown that the expression of PIK3R1 gene is decreased or lost in human cancers (Taniguchi et al., 2010; Urick et al., 2011; Cizkova et al., 2013), few researches explore the PIK3R1 gene in lung cancer. On the other hand, studies have demonstrated that PIK3R1 abrogation might reduce tumor proliferation and migration (Weber et al., 2011; Peng et al., 2013; Huang et al., 2015; Yan et al., 2016). This indicates that PIK3R1 gene may have different functions in diverse cancers. Therefore, cancer is an extremely complex disease and regulated by a lot of elements and biological processes. In this study, PIK3R1 was found to be upregulated as a potential oncogene in NSCLC.

Although miR-486-5p and PIK3R1 are respectively studied in various cancers, few studies investigate the regulative roles of miR-486-5p and PIK3R1 in lung cancer. However, miR-486-5p was reported to have different effects on the target PIK3R1 in A549 cell lines (Peng et al., 2013; Gao et al., 2018). Indeed, we identified several potential targets of miR-486-5p, including ST5 and SRSF3. The NCBI database shows that ST5 has the ability to suppress the tumorigenicity of Hela cells in nude mice. Although we selected PIK3R1 as a target to study here, there is no denying that the antioncogenic properties of miR-486-5p might not solely be explained by its ability to regulate a single gene because one miRNA could regulate numerous different genes in tumorigenesis. At the same time, single mRNA target could be also affected by numerous regulatory factors, including different miRNAs. Future studies are needed to identify and validate other targets of miR-486-5p. It will allow us to have deeper understanding underlying the cellular mechanism of development and progression in NSCLC.

Based on the results, it could be concluded that miR-486-5p might be involved in the *PI3K-Akt signaling pathway* by inhibiting PIK3R1 (**Supplementary Figure 2**). PI3K (PIK3CA and PIK3R1) produced PIP3 and eventually affected proliferation and antiapoptosis process (**Supplementary Figure 2**). Interestingly, the *PI3K-Akt signaling pathway* was involved in the *nonsmall cell lung cancer pathway* from the KEGG database (https://www.kegg.jp/). Moreover, EGFR and K-ras gene could affect PI3K gene in antiapoptosis, and PI3K might have an indirect effect on cell cycle and apoptosis in NSCLC. Therefore, the relations between miR-486-5p and PIK3R1 could be well applied in the *PI3K-Akt signaling pathway* and *nonsmall cell lung cancer pathway*.

In conclusion, our results revealed that miR-486-5p was significantly decreased in NSCLC tissue, serum, and cell samples as a diagnostic biomarker. Downregulated miR-486-5p was correlated with advanced stage and large tumor size in NSCLC, and low expression of miR-486-5p had significantly worse prognosis in lung SCC patients. The downregulation of miR-486-5p was negatively correlated with the upregulation of PIK3R1, a direct target gene, and overexpressed miR-486-5p inhibited cell proliferation and invasion in NSCLC. Overall, the results indicated that miR-486-5p might function as a diagnostic and prognostic biomarker, and a tumor suppressor in NSCLC therapy, and the findings could help us to better understand the mechanism of miRNA-related oncogenesis in lung cancer.

## Ethics Statement

This study was carried out in accordance with the recommendations of the Regulations and Guidelines on Ethical Review of Biomedical Clinical Research (trials) from the Ethics Committee of Jiangsu Province People’s Hospital. The protocol was approved by the Jiangsu Province People’s Hospital. All subjects gave written informed consent in accordance with the Declaration of Helsinki.

## Author Contributions

FT and YS performed the assay. TO and NL performed the computations and data analysis. FT and QG wrote the manuscript. JW and JL provided the clinical and cell samples. YB, XX, and QG conceived and designed the study.

## Conflict of Interest Statement

The authors declare that the research was conducted in the absence of any commercial or financial relationships that could be construed as a potential conflict of interest.

## Abbreviations

miRNA, MicroRNA; NSCLC, Nonsmall cell lung cancer; SCC, squamous cell carcinoma; AC, adenocarcinoma; UTR, untranslated region; CCK-8, Cell Counting Kit-8; NC, negative control; V-FITC, annexin V fluorescein isothiocyanate; PI, propidium iodide; siRNAs, small interfering RNAs.

## Supplementary Material

Supplementary Figure 1 The TCGA data showed miR-486-5p was significantly lower expressed in lung SCC (A, 322 samples, p < 0.01) and AC tissue (B, 430 samples, p < 0.01) compared to the controls.

Supplementary Figure 2 The PI3K-Akt signaling pathway and the regulatory relations of miR-486-5p and PIK3R1. The red line indicated the enhanced process or gene expression, and the green line indicated the attenuated process or inhibition. The dashed indicated the possible indirect effect on antiapoptosis, proliferation and metastasis.
