# Supplementary figures and images for: MiR-486-5p Serves as a Good Biomarker in Nonsmall Cell Lung Cancer and Suppresses Cell Growth With the Involvement of a Target PIK3R1
Source: Front Genet. 2019 Jul 26;10:688. doi: 10.3389/fgene.2019.00688 (PMC6675869; doi:10.3389/fgene.2019.00688)

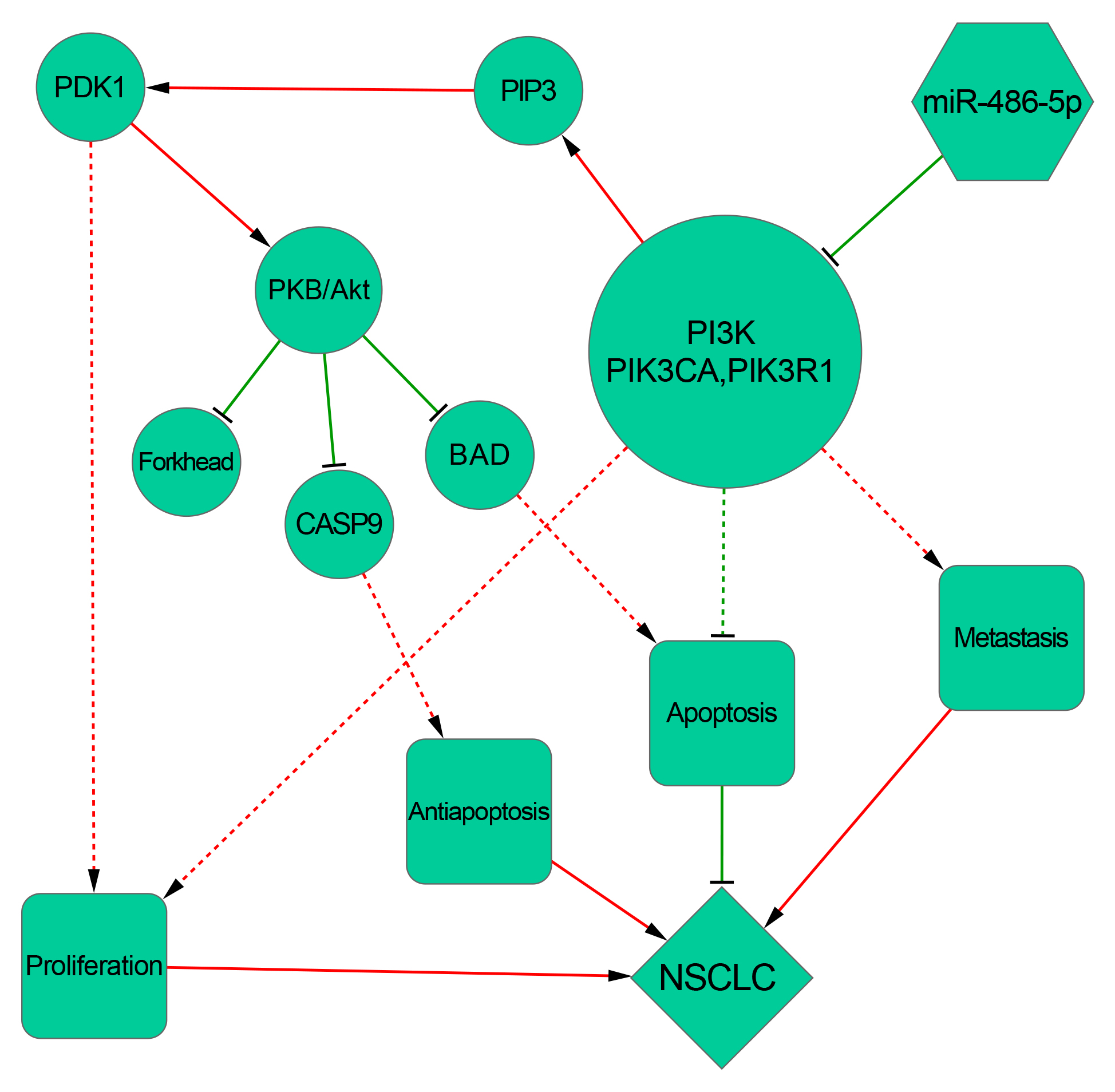

Supplement: Supplementary Figure 2 — The PI3K-Akt signaling pathway and the regulatory relations of miR-486-5p and PIK3R1. The red line indicated the enhanced process or gene expression, and the green line indicated the attenuated process or inhibition. The dashed indicated the possible indirect effect on antiapoptosis, proliferation and metastasis. [file Image_2.tif]
