# Supplementary material for: MiR-486-5p Serves as a Good Biomarker in Nonsmall Cell Lung Cancer and Suppresses Cell Growth With the Involvement of a Target PIK3R1
Source: Front Genet. 2019 Jul 26;10:688. doi: 10.3389/fgene.2019.00688 (PMC6675869; doi:10.3389/fgene.2019.00688)
Supplement: Supplementary file 3 [file Table_1.docx]

**Supplementary Tables**

Supplementary Table1 The demographic and clinical data of 39 NSCLC patients, and the associations with miR-486-5p expression in tumor tissue

| **Tumor characteristics** | **Number of patients (%)** | **MiR-486-5p expression**  **(mean ΔCt ± SD)** | **P-value** |
| --- | --- | --- | --- |
| *Age (year)* |  | | |
| ≥ 60 | 20 (51.3) | 11.3688±1.3171 | 0.3414 |
| < 60 | 19 (48.7) | 11.4105±3.7851 |  |
| *Gender* |  | | |
| Male | 18 (46.2) | 11.3419±1.2548 | 0.5542 |
| Female | 21 (53.8) | 11.4296±3.6333 |  |
| *Smoking state* |  | | |
| Yes | 23 (59.0) | 11.7329±1.9072 | 0.2215 |
| No | 16 (41.0) | 10.8949±3.6897 |  |
| *Histology type* |  | | |
| Squamous cell carcinoma | 22 (56.4) | 11.8245±1.5811 | 0.1529 |
| Adenocarcinoma | 17 (43.6) | 10.8256±3.7814 |  |
| *Stage* |  | | |
| I | 12 (30.8) | 8.2851±1.1443 | < 0.001 |
| II | 13 (33.3) | 11.0707±0.9096 |  |
| III | 14 (35.9) | 14.3453±1.4476 |  |
| *Tumor Size* |  | | |
| ≤ 30mm | 14 (35.9) | 8.5159±1.2051 | < 0.001 |
| 30-50mm | 15 (38.5) | 11.6983±1.0224 |  |
| ≥ 50mm | 10 (25.6) | 14.9477±1.2338 |  |

Supplementary Table2 The primer sequence used in the qRT-PCR assay

| **Primer Name** | | **Sequence (5’-3’)** |
| --- | --- | --- |
| miR-486-5p RTP | GTCGTATCCAGTGCAGGGTCCGAGGTATTCGCACTGGATACGACCTCGGG | |
| miR-486-5p F | TCCTGTACTGAGCTGCCC | |
| miRNA universal R | GTGCAGGGTCCGAGGT | |
| U6 RTP | GCTTCACGAATTTGCGTGTCAT | |
| U6 F | GCTTCGGCAGCACATATACTAAA | |
| U6 R | GCTTCACGAATTTGCGTGTCAT | |
| mRNA RTP | NNNNNN | |
| PIK3R1 F | ACCACTACCGGAATGAATCTCT | |
| PIK3R1 R | GGGATGTGCGGGTATATTCTTC | |
| β-Actin F | CATGTACGTTGCTATCCAGGC | |
| β-Actin R | CTCCTTAATGTCACGCACGAT | |

* RTP: reverse transcription primer; F: forward primer; R: reverse primer.

Supplementary Table3 The sequences used in the report vector, lentivector and RNA silencing

| **Primer Name** | **Sequence (5’-3’)** |
| --- | --- |
| PIK3R1 WT S | CTGTTAATATTCATTTTTGTTATCCTTTTTTAAAAGTAAATGTACAGGATGCCAGTAAAAAAAAAAAATGGC |
| PIK3R1 WT A | TCGAGCCATTTTTTTTTTTTACTGGCATCCTGTACATTTACTTTTAAAAAAGGATAACAAAAATGAATATTAACAGAGCT |
| PIK3R1 MUT S | CTGTTAATATTCATTTTTGTTATCCTTTTTTAAATCTAAATCATGTCCATGCCAGTAAAAAAAAAAAATGGC |
| PIK3R1 MUT A | TCGAGCCATTTTTTTTTTTTACTGGCATGGACATGATTTAGATTTAAAAAAGGATAACAAAAATGAATATTAACAGAGCT |
| miR-486-5p PC S | CCTCGGGGCAGCTCAGTACAGGAACCGGTCTCGGGGCAGCTCAGTACAGGAC |
| miR-486-5p PC A | TCGAGTCCTGTACTGAGCTGCCCCGAGACCGGTTCCTGTACTGAGCTGCCCCGAGGAGCT |
| miR-486-5p mimic | UCCUGUACUGAGCUGCCCCGAG |
| mimic NC | UUCUCCGAACGUGUCACGUTT |
| pre-miR-486-5p S | CCGGGCATCCTGTACTGAGCTGCCCCGAGGCCCTTCATGCTGCCCAGCTCGGGGCAGCTCAGTACAGGATACG |
| PIK3R1 si-739 | GCUGGUUAAAUGGCUAUAATT |
| PIK3R1 si-1178 | GCAGCCGUUUACAGUGAAATT |
| miR-486-5p inhibitor | CUCGGGGCAGCUCAGUACAGGA |

* WT: wild type; MUT: mutation type; S: sense strand; A: antisense strand; PC: positive control; NC: negative control.
